# Increase in sensitization to common airborne allergens among adults – two population-based studies 15 years apart

**DOI:** 10.1186/1710-1492-9-20

**Published:** 2013-06-11

**Authors:** Katja Warm, Anne Lindberg, Bo Lundbäck, Eva Rönmark

**Affiliations:** 1 The OLIN studies, Norrbotten County Council, Robertsviksgatan 9, Luleå, S-971 89, Sweden; 2 Department of Respiratory Medicine and Allergology, Sunderby Central Hospital of Norrbotten, Luleå, Sweden; 3 Department of Public Health and Clinical Medicine, Umeå University, Umeå, Sweden; 4 Institute of Medicine/Krefting Research Center, University of Gothenburg, Gothenburg, Sweden

**Keywords:** Allergic sensitization, Epidemiology, IgE, Population study, Prevalence, Skin prick test

## Abstract

**Background:**

Studies on time trends of allergic sensitization among adults are rare. The aim of the study was to compare the prevalence of allergic sensitization to common airborne allergens among adults 15 years apart and to identify risk factors for allergic sensitization.

**Methods:**

Clinical examinations including skin prick test (SPT) and structured interviews were performed in two random population samples in 1994 and 2009. Furthermore, specific IgE was analyzed in 2009. SPT data were available for 483 subjects in 1994 and for 463 subjects in 2009 in ages 20–60 years. Specific IgE was analyzed in 692 subjects in ages 20–79 years.

**Results:**

Sensitization to cat (16% to 26%, p < 0.001), dog (13% to 25%, p < 0.001), birch (13% to 18%, p = 0.031) and timothy (12% to 21%, p < 0.001), based on SPT, increased significantly from 1994 to 2009. Sensitization to any positive SPT increased from 35% to 39%, p = 0.13.The proportion of having ≥3 positive SPT reactions increased from 40% to 56%, p = 0.002. The sensitization pattern yielded similar results based on specific IgE. Risk factors for allergic sensitization were having a family history of allergy (OR 3.1, 95% CI 2.0-4.8 for any positive SPT; OR 2.7, 95% CI 1.8-4.0 for any elevated IgE) and urban living (OR 1.7, 95% CI 1.0-2.7; OR 1.5, 95% CI 1.0-2.4).

**Conclusions:**

The prevalence of allergic sensitization to major airborne allergens as well as multi-sensitization increased significantly between the study years. Young age, a family history of allergy and urban living were significant risk factors for allergic sensitization.

## Background

In contrast to studies among children, few studies based on objective clinical methods have analyzed time trends in prevalence of allergic sensitization among adults. Studies performed during the 1990s have reported an increasing prevalence of sensitization [1-3]. However, it is not clear if this trend of an increase is continuing. Recent studies among children and adolescents suggest that the earlier reported increase in prevalence of allergic sensitization over several decades might have levelled off [4,5], while others report an on-going increase [6,7]. Furthermore, trends in prevalence of sensitization to specific allergens have been sparsely studied among both children and adults.

The major risk factor for allergic sensitization is a family history of allergic diseases [7,8], while the impact of lifestyle factors is still debated. Several studies emphasize the importance of living conditions and exposures in early childhood. The presence of furry animals at home in childhood is associated with a decreased risk for developing allergic sensitization [7,9]. Similarly, a negative association has also been found for farm or rural living and having several siblings [9-11]. Environmental influences like increased urbanization and increase in air pollution have probably contributed to the increase in prevalence of allergic sensitization [12]. Furthermore, gene-environment interactions are of importance [13].

The aim of this study was to compare the prevalence of allergic sensitization to common airborne allergens among adults in 1994 to 2009 in Northern Sweden and to identify risk factors for allergic sensitization.

## Material & methods

Two cross-sectional studies using identical methods were performed within the Obstructive Lung Diseases in Northern Sweden (OLIN) studies 15 years apart, 1994 and 2009. The area covers 25% of the area of Sweden, has cold and dry winters and is almost mite-free [14]. The studies were approved by the Regional Ethical Review Board at Umeå University, Sweden.

### Study population

In 1992 a postal questionnaire was mailed to 5,682 randomly selected subjects aged 20–69 years in Norrbotten, and 85% responded. A random sample of 970 questionnaire responders was invited to clinical examinations in 1994 including structured interview and skin prick testing (SPT), and 664 (68.5%) subjects participated. SPT was performed in subjects ≤ 60 years (n = 483, 51% women) [15].

In 2006 another randomly selected cohort in ages 20–69 years was recruited, n = 7,997. A follow-up of a randomly selected cohort recruited in 1996 was also performed, now aged 30–79 years, n = 7,004. Of all invited, 12,055 subjects (80%) participated in a postal questionnaire survey. Of the responders, 1,000 subjects were randomly selected after stratification for the age and sex distribution of the general population in the study area. In 2009 they were invited to clinical examinations, and 737 (73.7%) subjects participated. SPT was performed in 463 subjects ≤60 years (50% women). Of these 737 subjects, 378 came from the follow-up cohort from 1996 and 359 from the newly recruited cohort. Blood samples for total and specific IgE were collected from 692 subjects (51% women), or 94% of all participants. In 1994, the mean age among the participants in skin prick testing was 44.1 years (SD 9.73), in 2009 it was 44.6 years (SD 10.54), p = 0.436. Table 1 shows the demography of the SPT participants in the two cohorts.

**Table 1:** Demography of participants in the SPT in 1994 and 2009

	**Study year**		
	**1994**	**2009**	**p-value**
	n = 483	n = 463	
Mean age (SD)	44.05 (9.73)	44.57 (10.54)	0.436
Men, n (%)	237 (49.1)	233 (50.3)	0.699
Age group, n (%)			
20-29 y	47 (9.7)	56 (12.1)	0.081
30-39 y	109 (22.6)	87 (18.8)	
40-49 y	163 (33.7)	135 (29.2)	
50-60 y	164 (34.0)	185 (40.0)	
Family history of allergic rhinitis (%)	33.9	32	0.544
Ever smoker (%)	57.1	45.6	<0.001
Furry animals in cildhood (%)	77.5	63.7	<0.001
Urban living in childhood (%)	32.5	54.5	<0.001
Urban living (%)	68.1	72.2	0.174

### Questionnaire

The OLIN questionnaire was used in both surveys, and it has also been used in several international studies [8,16,17]. It has been externally validated against the GA2LEN questionnaire [18]. The questions are focused on respiratory symptoms and diseases, family history of asthma and allergic diseases, smoking habits and occupation. The interview questionnaire was an expanded version of the postal questionnaire.

### Skin prick test

The test included a panel of ten common airborne allergens: birch, timothy, mugwort, cat, dog, horse, D. pteronyssinus, D. farinae, cladosporium and alternaria. Experienced nurses performed the tests with identical methods in both studies, and according to international guidelines [19] as single-tests on one fore-arm with lancets and standardized allergens (Solo-Prick, ALK, Denmark). A wheal size ≥3 mm was regarded as a positive reaction. Histamine and glycerol were used as positive and negative control, respectively. All subjects included in the analysis had a positive reaction to histamine and none of the subjects had reacted to the negative control.

#### IgE

Blood samples for specific IgE to birch, timothy, mugwort, cat, dog, horse, D. pteronyssinus, D. farinae and alternaria were collected in the 2009 survey. The serum samples were stored at −20°C and analyzed with the Immuno CAP system (ThermoFisher, Uppsala, Sweden). A positive result was defined as an IgE-level ≥0.35 IU/ml.

### Definitions

*Any positive SPT:* At least one positive SPT reaction.

*Any elevated IgE*: A level of IgE ≥0.35 IU/ml to any specific allergen.

*Multi-sensitization SPT (IgE)*: ≥2 positive SPT reactions (elevated specific IgE).

*Family history of allergy*: “Have any of your parents or siblings had allergic eye-/nose catarrh (hay fever)?”

*Furry animals in childhood*: “Were there any furry animals or birds at home in your early childhood?”

*Urban living*: Living in a town ≥ 2000 inhabitants.

*Socio-economic status* was classified based on occupation defined by Statistics Sweden.

*Smoking habits*: Smokers were currently smokers or had smoked during the year preceding the examination. Ex-smokers had smoked for at least one year but not during the last 12 months. Non-smokers had never smoked. Smokers and ex-smokers were classified as ever smokers.

### Statistical analyses

The Statistical package for Social Sciences (SPSS) for Windows, Version 18.0, was used for statistical analysis. Chi square test, and Fisher´s exact test when appropriate, was used for comparison of categorical variables, and student *t*-test for comparison of continuous variables. A p-value <0.05 was considered as statistically significant. Multiple logistic regression analyses were used to calculate risk factors for allergic sensitization in 2009 and risks were expressed as odds ratios (OR) with 95% confidence intervals (CI). Dependent variables were “any pollen”, “any animal” and “any allergen” according to SPT and specific IgE. Sex and significant or borderline significant (p < 0.1) risk factors for any elevated IgE in bi-variate analyses were included as independent variables in the multivariate models. Risk factors for multi-sensitization versus non-sensitization were also analyzed.

## Results

### Prevalence of allergic sensitization

**Figure 1:**
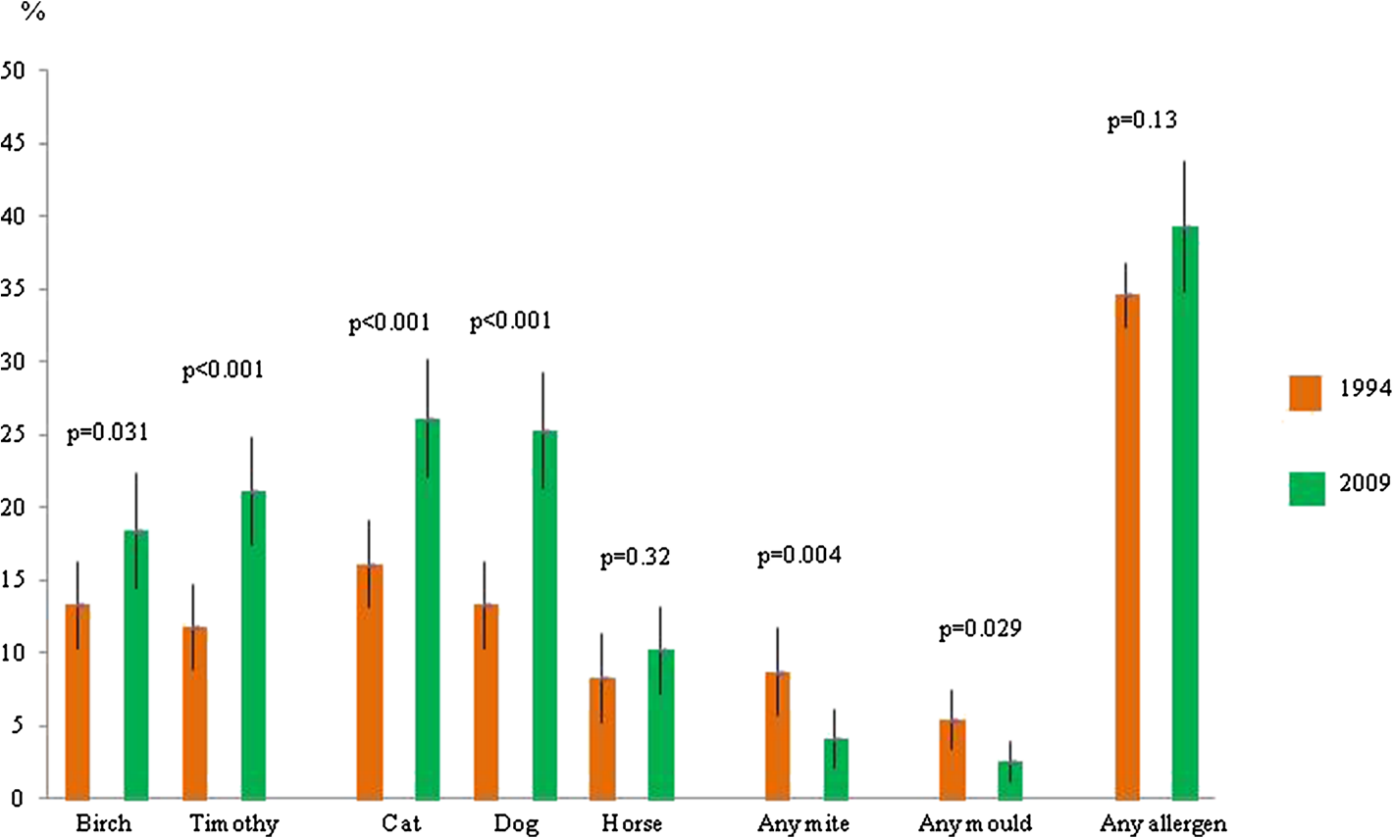
**Prevalence (%) and 95% confidence intervals of positive skin prick test to the most common allergens by study year.** Difference (p-value) by study year.

**Figure 2:**
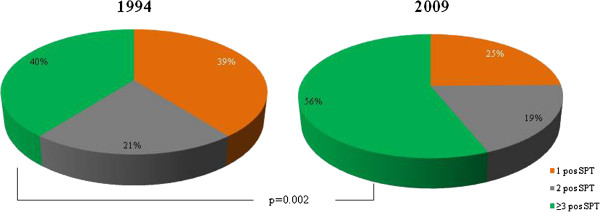
**Proportions (%) of having one, two or at least three positive SPT among all sensitized subjects in 1994 and 2009, respectively.** Difference (p-value) by study year.

**Figure 3:**
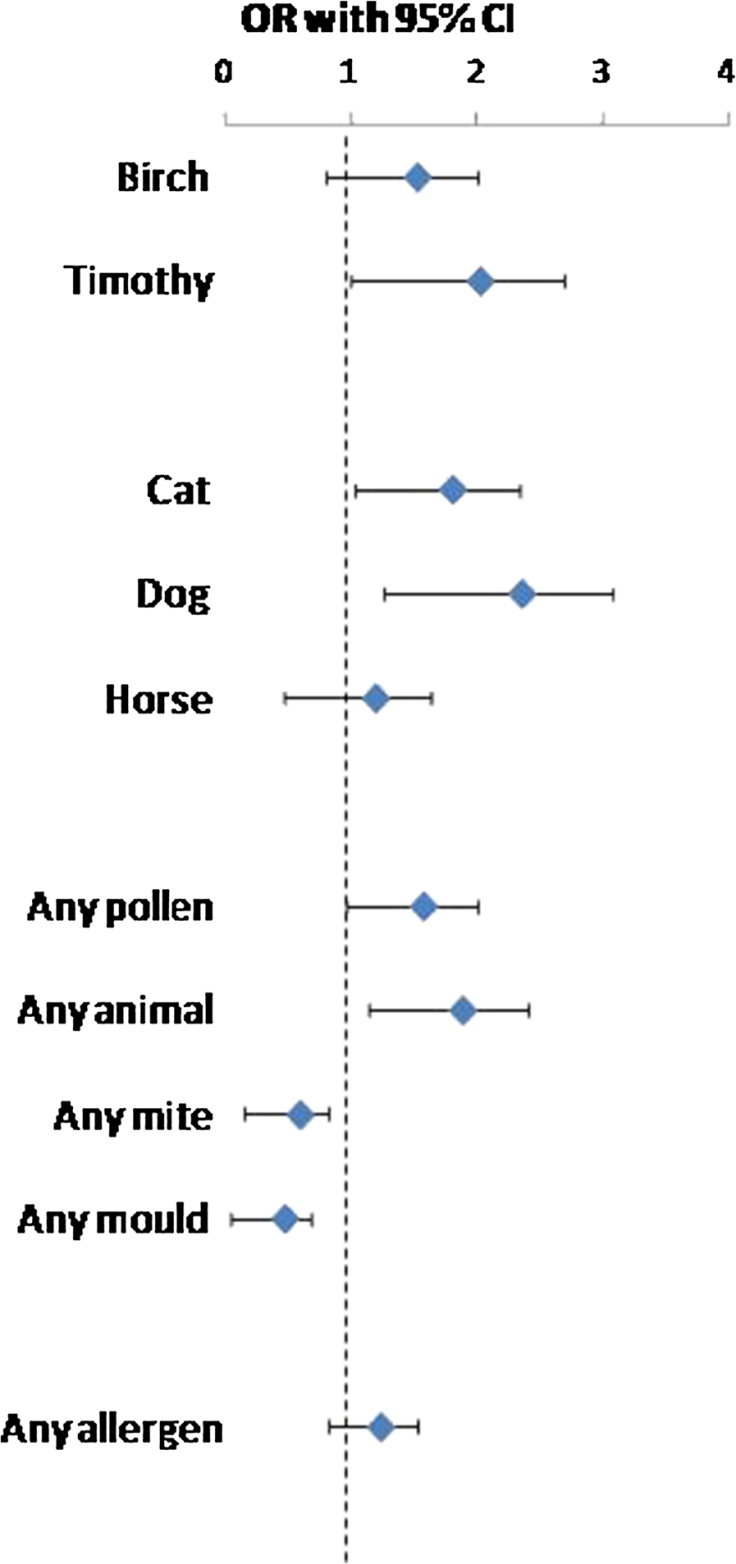
**Increase in sensitization to common airborne allergens between 1994 and 2009 expressed as ORs.** Analyzed by multiple logistic regression analysis adjusted for sex, age, family history of allergy, ever smoking, urban living in childhood and the presence of furry animals in childhood.

The prevalence of a positive SPT to birch, timothy, cat and dog, respectively, increased significantly between 1994 and 2009 (Figure 1). The prevalence of any positive SPT increased from 34.6% (95% CI 32.4-36.8%) to 39.3% (34.8-43.8%), however not significantly (p = 0.13). A significant increase in prevalence of multi-sensitization was found (Figure 2). When corrected for potential confounders, the increasing trends in sensitization between the surveys were confirmed (Figure 3).

The sensitization patterns were similar in both surveys. Most common was sensitization to cat (16.1% in 1994; 26.1% in 2009, p < 0.001), followed by dog (13.3%; 25.3%, p < 0.001), birch (13.3%; 18.4%, p = 0.031), and timothy (11.8%; 21.2%, p < 0.001) (Figure 1). Sensitization to mites and moulds was uncommon both years (Figure 1; Table 2). In both surveys a positive SPT to any allergen was most common in the youngest age group, 20–29 years (55.3%; 60.7%, p = 0.58) and lowest in the oldest age group, 50–60 years (26.2%; 28.6% p = 0.61). A decrease in prevalence by age was also found for sensitization to birch, timothy, cat, and dog (Table 2).

**Table 2:** Prevalence (%) of positive skin prick test by age group and sex in 1994 and 2009, respectively; difference (p-value) by study year

		**Age group**								**Sex**			
**Allergen**		**20-29 y**	**p-value**	**30-39 y**	**p-value**	**40-49 y**	**p-value**	**50-60 y**	**p-value**	**Men**	**p-value**	**Women**	**p-value**
Birch	1994	23.4	0.124	19.3	0.804	13.5	0.068	6.1	0.281	13.5	0.089	13.0	0.182
	2009	37.5		20.7		21.5		9.2		19.3		17.4	
Timothy	1994	27.7	0.050	17.4	0.562	8.6	0.002	6.7	0.037	13.9	0.024	9.8	0.001
	2009	46.4		20.7		21.5		13.5		21.9		20.4	
Mugwort	1994	2.1	0.398	0.9	0.051	8.0	0.028	1.2	0.012	3.8	0.620	3.3	0.286
	2009	5.4		5.7		2.2		6.5		4.7		5.2	
Cat	1994	23.4	0.060	22.9	0.271	15.3	0.016	10.4	0.018	16.9	0.001	15.4	0.060
	2009	41.4		29.9		26.7		19.5		30.0		22.2	
Dog	1994	17.0	0.005	20.2	0.301	12.3	0.001	8.5	0.016	13.5	<0.001	13.0	0.012
	2009	42.9		26.4		32.5		17.3		28.8		21.7	
Horse	1994	14.9	0.288	13.8	0.636	8.6	0.755	2.4	0.107	8.9	0.329	7.7	0.699
	2009	23.2		11.5		9.6		5.9		11.6		8.7	
D.pteronyssinus	1994	2.1	0.142	10.1	0.029	4.9	0.851	5.5	0.048	5.9	0.038	6.1	0.528
	2009	8.9		2.3		4.4		1.6		2.1		4.8	
D.farinae	1994	8.5	0.940	5.5	0.026	4.3	0.544	5.5	0.006	5.5	0.109	5.3	0.037
	2009	8.9		0		3.0		0.5		2.6		1.7	
Cladosporium	1994	8.5	0.526	2.8	0.430	2.5	0.552	3.0	0.191	3.0	0.375	3.7	0.199
	2009	5.4		1.1		1.5		1.1		1.7		1.7	
Alternaria	1994	4.3	0.858	3.7	0.266	4.3	0.158	1.8	0.065	4.6	0.032	2.0	0.292
	2009	3.6		1.1		1.5		0		1.3		0.9	
Any allergen	1994	55.3	0.580	36.7	0.504	35.6	0.153	26.2	0.612	34.2	0.078	35.0	0.722
	2009	60.7		41.4		43.7		28.6		42.1		36.5	

The mean wheal sizes differed slightly between the study years: birch 6.1 mm in 1994 versus 4.7 mm in 2009 (p < 0.001) and dog with 6.0 mm versus 4.9 mm (p = 0.004). They were similar for timothy with 6.2 mm versus 5.8 mm and for cat with 7.3 mm versus 6.6 mm, respectively. The reaction to histamine was slightly larger in 1994 compared to 2009, 6.5 mm versus 5.7 mm (p < 0.001).

The prevalence of allergic sensitization based on specific IgE in 2009 showed a similar pattern compared to the SPT results. In general, the prevalence of allergic sensitization was somewhat lower based on specific IgE compared to SPT (data not shown).

### Risk factors for allergic sensitization in 2009

Young age, a family history of allergy and urban living were significantly associated with elevated IgE to any allergen in bi-variate analyses. Ever smoking was significantly negatively associated with elevated IgE to any allergen, as was having had furry animals in childhood and growing up on a farm. No associations were found between elevated IgE to any allergen and number of older siblings, maternal smoking in childhood or socio-economic status.

In the multivariate models, increasing age was strongly associated with a lower risk for any elevated IgE. Other significant risk factors were a family history of allergy, OR 2.68 (95% CI 1.80-3.97) and urban living, OR 1.54 (1.00-2.36). Urban living was associated with an increased risk for elevated specific IgE to any pollen, OR 1.79 (1.06-3.03). Having had furry animals in childhood was associated with a reduced risk for elevated specific IgE to any animal, OR 0.62 (0.39-0.99) (Table 3). When added to the statistical model, the variable growing up on a farm lost its significance as it was strongly related to age and very few young adults had grown up on a farm. Consequently, when age was excluded from the model, growing up on a farm became significantly negatively related to any elevated IgE, OR 0.62 (0.41-0.94).

**Table 3:** Risk factors for positive skin prick test (SPT) and elevated specific IgE in 2009 expressed as odds ratios (OR) with 95% confidence intervals (CI) calculated by multiple logistic regression analysis

	**SPT**						**Specific IgE**					
	**Any pollen**		**Any animal**		**Any SPT**		**Any pollen**		**Any animal**		**Any IgE**	
**Independent variable**	**OR**	**95% CI**	**OR**	**95% CI**	**OR**	**95% CI**	**OR**	**95% CI**	**OR**	**95% CI**	**OR**	**95% CI**
Age												
20-29 y	1	-	1	-	1	-	1	-	1	-	1	-
30-39 y	0.48	0.23-1.00	0.57	0.27-1.19	0.55	0.26-1.14	0.44	0.20-0.95	0.93	0.40-2.13	0.55	0.26-1.17
40-49 y	0.54	0.27-1.07	0.69	0.35-1.36	0.67	0.34-1.33	0.49	0.24-0.98	0.96	0.45-2.06	0.62	0.31-1.22
50-60 y	0.31	0.15-0.62	0.46	0.23-0.91	0.39	0.20-0.77	0.29	0.14-0.60	0.65	0.29-1.44	0.39	0.19-0.78
>61 y							0.10	0.05-0.25	0.32	0.13-0.74	0.20	0.10-0.40
Male sex	1.38	0.88-2.14	1.69	1.09-2.63	1.51	1.00-2.29	1.30	0.85-2.01	1.40	0.90-2.19	1.30	0.90-1.87
Family history of allergy	3.16	2.01-4.97	3.26	2.07-5.12	3.12	2.02-4.83	2.68	1.72-4.20	3.13	1.98-4.94	2.68	1.80-3.97
Ever smoked	0.89	0.57-1.39	0.79	0.51-1.23	0.93	0.62-1.42	0.69	0.44-1.06	0.78	0.50-1.22	0.79	0.55-1.14
Urban living	1.58	0.95-2.64	1.56	0.94-2.60	1.66	1.03-2.66	1.79	1.06-3.03	1.14	0.69-1.88	1.54	1.00-2.36
Urban living in childhood	0.90	0.56-1.43	1.06	0.67-1.68	1.08	0.70-1.67	1.05	0.66-1.67	0.87	0.54-1.41	0.87	0.58-1.31
Furry animals in childhood	0.81	0.51-1.28	0.80	0.51-1.25	0.82	0.53-1.26	0.96	0.60-1.49	0.62	0.39-0.99	0.79	0.53-1.18

The risk factor pattern was similar when based on SPT. Significant risk factors were young age, a family history of allergy and urban living. Male sex was associated with an increased risk for any positive SPT and a positive SPT to any animal (Table 3).

Risk factor analyses for multi-sensitization yielded similar results, although a family history of allergy appeared as an even stronger risk factor, OR 3.89 (95% CI 2.41-6.28) and OR 3.39 (2.13-5.41), based on SPT and specific IgE, respectively.

## Discussion

We compared the prevalence of allergic sensitization among adults in two cross-sectional population based studies performed in 1994 and 2009, and found a significant increase in prevalence of sensitization to the major airborne allergens cat, dog, birch and timothy. The prevalence of multi-sensitization increased also significantly, while the increase of sensitization to at least one allergen, from 35% to 39%, did not reach significance.

Only a few studies have reported time trends of allergic sensitization among adults [1-3,20,21]. A Danish study investigated time trends in allergic sensitization over 25 years at three occasions using specific IgE in subjects aged 30 to 60 years. In line with our results, they found an increase in prevalence of allergic sensitization in all age groups, but the study did not report on trends in sensitization to specific allergens or changes in the sensitization pattern [2]. Similarly, a study from Finland found an increase in prevalence of allergic sensitization among adults during the last two decades of the previous century [3]. The US NHANES, comparing positive SPT-reactions in two large surveys from 1976–1980 to 1988–1994, found an increase in the overall prevalence from 22% to 42% [21]. However, the NHANES study population included both children and adults, and there were methodological differences between the two surveys.

Our study is the first to report time trends in allergic sensitization among adults in Sweden during this century and suggests that the prevalence is still increasing. This is consistent with results from a study among children carried out in the same area during the same period [7]. However, as we examined allergic sensitization in two adult populations, and sensitization may have occurred at young age or during adolescence, our results in fact reflect prevalence trends two or three decades before the studies were performed.

We found a similar sensitization pattern both years with cat and dog being the most common allergens followed by pollen from birch and timothy, confirming results from earlier Scandinavian studies [8,22]. The prevalence of allergic sensitization measured by specific IgE in 2009 yielded slightly lower results than by using SPT, especially for cat and dog, but confirmed the sensitization pattern. Several international studies have found house dust mite to be the major airborne allergen [17,23]. In Northern Sweden, however, mites are uncommon due to the cold and dry climate [14,22].

Very few studies comparing time trends in prevalence of mono- and multi-sensitization have been published so far. Linneberg et al. compared sensitization to more than one allergen assessed by specific IgE between 1990 and 1998. In line with our results, they found a significant increase in multi-sensitization [20]. The impact of multi-sensitization on allergic clinical conditions has been described earlier [8,24,25], and the number of positive SPT responses correlates with bronchial hyper reactivity [26]. It will be of further interest to study the impact of the increase in multi-sensitization on trends in prevalence of allergic diseases.

Young age, a family history of allergy and urban living were significantly associated with allergic sensitization. This is concordant with earlier published data [3,8,15,17]. The higher prevalence of allergic sensitization among young compared to older adults is well-known [27]. The decrease in prevalence by age among adults could be related to a birth-cohort-effect. However, we have shown that the decrease in allergic sensitization with increasing age is mainly influenced by normal ageing [15].

Several studies among children have found that having pets at home is associated with a reduced risk of sensitization [7,28]. In this study, having had pets at home in childhood was negatively associated with an elevated level of specific IgE to pets. A Finnish study reported that contact with farm animals, and also cats and dogs, reduced the risk of not only allergic sensitization but also of asthma and other allergic clinical conditions [29].

Growing up in a rural environment has been reported to be a protective factor against allergic sensitization [29,30]. The Canadian Humboldt Study found a lower prevalence of allergic sensitization among subjects who currently lived on a farm [31]. We found rural living in childhood, as well as growing up on a farm, to be significantly negatively associated with elevated IgE in bivariate analyses. This effect could not be verified in the multivariate analyses as only few young adults had grown up on a farm compared to more than 50% among the elderly. Our sample size had not power enough for allowing stratified analyses by age group. However, farming becoming uncommon parallel with urbanization may have contributed to the increase of allergic sensitization also in our study area.

In bi-variate analysis we found a negative association between smoking and allergic sensitization, however this effect disappeared in the multivariate model. Some studies have reported a protective effect of smoking on allergic sensitization [15,32,33], while others did not find any significant association [8]. It has been shown that tobacco smoke inhibits pro-inflammatory cytokines and thus has an immunosuppressive effect [34]. Further experimental studies are needed to clarify the role of smoking on the immune system related to allergic sensitization.

We observed a significant positive association between urban living and allergic sensitization. This relationship was strongest for specific IgE to pollen. It has been shown that air pollution influences the allergenic potential of pollen, and a higher pro-inflammatory and chemotactic activity has been observed in pollen collected near heavy-traffic roads [35-37]. Further, pollutants related to heavy traffic increase the secretion of cytoplasmatic granules in timothy pollen [38]. The European Community Respiratory Health Survey compared fine particle mass and sulphur exposure with the prevalence of allergic sensitization and found a positive, however not significant, association between air pollutants and allergic sensitization [39].

Parallel to the increase in prevalence of allergic sensitization some life style factors had changed significantly. Compared with the cohort from 1994, fewer subjects in the recent cohort had grown up in rural areas. Smoking was also less prevalent in the recent cohort. These changes in life style may have contributed to the observed increase in prevalence of allergic sensitization.

The strength of our study is the identical age and gender distribution in the two study samples. The study population in 2009 consisted of subjects from a follow-up study of a cohort recruited in 1996 and a newly recruited cohort. These two sub samples were almost identical regarding the SPT results, the prevalence of asthma, respiratory symptoms, use of asthma medication and smoking. Thus, the design of the cohort from 2006 did not bias the results. Validated and internationally established questionnaires were used to collect data on risk factors. Further, we were able to provide data both on SPT and specific IgE, confirming the sensitization pattern. The somewhat lower prevalence when measuring allergic sensitization by using specific IgE has been shown earlier [15,40]. Regarding skin prick testing there is a risk of method bias. However, only a small number of experienced staff performed the SPTs, and identical methods with solutions from the same manufacturer were used in both surveys. For some of the tested allergens the mean wheal diameter was slightly lower in 2009 compared to 1994. Thus, the wheal sizes may have been overestimated in 1994 or underestimated in 2009, and would accordingly strengthen our results of an increasing prevalence of allergic sensitization over the 15 years.

In conclusion, from 1994 to 2009 the prevalence of allergic sensitization to the major allergens in the studied area, as well as multi-sensitization, increased significantly among adults. The overall prevalence of sensitization to at least one allergen increased also, however not significantly. Young age and a family history of allergy were the most important risk factors. Additionally, urban living was associated with an increased risk for allergic sensitization, especially to pollen. Our study results of an on-going increase of allergic sensitization emphasizes the need for further studies of environmental risk factors as well as clinical effects of the increased prevalence.

## Abbreviations

OLIN: Obstructive lung diseases in Northern Sweden; SPT: Skin prick test; SD: Standard deviation; OR: Odds Ratio; CI: Confidence interval; GA2LEN: Global Allergy and Asthma European Network; NHANES: National Health and Nutrition Examination Survey.

## Competing interests

None of the authors has any conflicts of interests to disclose.

## Authors’ contributions

KW contributed to the design of the study, participated in data collection, performed statistical analyses and drafted the manuscript. AL participated in analysing and interpreting of data and helped draft the manuscript. BL contributed to the design of the study, participated in analysing and interpreting of data and helped draft the manuscript. ER designed the study, participated in data collection, participated in analysing and interpreting of data and helped draft the manuscript. All authors read and approved the final manuscript.
